# The Impact of the COVID-19 Pandemic on Trajectories of Well-Being of Middle-Aged and older Adults: A Multidimensional and Multidirectional Perspective

**DOI:** 10.1007/s10902-022-00552-z

**Published:** 2022-08-23

**Authors:** Markus Wettstein, Hans-Werner Wahl, Anna Schlomann

**Affiliations:** 1 Network Aging Research, Heidelberg University, Heidelberg, Germany; 2 Institute of Psychology, Heidelberg University, Heidelberg, Germany; 3 Institute for Educational Sciences, Heidelberg University of Education, Heidelberg, Germany; 4 Humboldt-Universität zu Berlin, Berlin, Germany

**Keywords:** Eudaimonic well-being, Hedonic well-being, Life satisfaction, Corona crisis, Midlife, Old age

## Abstract

The COVID-19 pandemic has resulted in profound changes of individuals’ everyday lives. Restrictions in social contacts and in leisure activities and the threatening situation of a spreading virus might have resulted in compromised well-being. At the same time, the pandemic could have promoted specific aspects of psychosocial well-being, e.g., due to intensified relationships with close persons during lockdown periods. We investigated this potentially multidimensional and multi-directional pattern of pandemic-specific change in well-being by analyzing changes over up to 8 years (2012-2020) in two broad well-being domains, hedonic well-being (life satisfaction) and eudaimonic well-being (one overarching eudaimonic well-being indicator as well as environmental mastery, personal growth, positive relations with others, and self-acceptance), among 423 adults who were aged 40-98 years in 2012. By modelling longitudinal multilevel regression models and allowing for a measurement-specific intra-individual deviation component from the general slope in 2020, i.e. after the pandemic outbreak, we analyzed potential normative history-graded changes due to the pandemic. All mean-level history-graded changes were nonsignificant, but most revealed substantial interindividual variability, indicating that individuals’ pandemic-related well-being changes were remarkably heterogeneous. Only for personal growth and self-acceptance, adding a pandemic-related change component (and interindividual variability thereof) did not result in a better model fit. Individuals with poorer self-rated health at baseline in 2012 revealed a pandemic-related change toward lower life satisfaction. Our findings suggest that not all well-being domains - and not all individuals - are equally prone to “COVID-19 effects”, and even pandemic-associated gains were observed for some individuals in certain well-being domains.

The outbreak of the still ongoing Corona pandemic had dramatic consequences for the everyday lives of societies and individuals (Settersten et al., 2020). Specifically, individuals were obliged or at least encouraged, due to governmental rules and recommendations, to contribute to slowing down the spreading of the virus by reducing their face-to-face social contacts as well as their out-of-home activities. Leisure options were considerably limited as long as facilities such as cinemas, gyms or restaurants were closed and cultural events were cancelled. Many individuals, particularly middle-aged adults, had to re-organize their work and family life by working from home and taking care of their children as long as schools and childcare facilities were closed. In addition, the threatening situation of a spreading, potentially dangerous virus, with heightened COVID-19-related health risks particularly among older adults (Karagiannidis et al., 2020; Nachtigall et al., 2020; Robert-Koch-Institut, 2020) might have caused fears, in particular “virus anxiety” (Jungmann & Witthöft, 2020) or “COVID-19 phobia” (Petzold et al., 2020) and psychological distress among many individuals.

The aim of this study is therefore to investigate if and to what extent individuals’ well-being has changed after the onset of the pandemic in Germany. We address this research question by focusing on middle-aged and older adults and by investigating well-being changes across eight years (2012–2020), over a time interval comprising both several pre-pandemic measurement occasions (2012, 2015, and 2017) as well as one peri-pandemic measurement occasion that took place between June and September 2020. We adopt the macro-model of developmental influences (Baltes et al., 2006). This model specifies *normative age-graded influences on development*, i.e. “biological and environmental aspects that, because of their dominant age correlation, shape individuals in relatively normative ways for all individuals” (p. 586). An additional influence component according to this model are *normative history-graded influences on development*, which are “biological and environmental aspects that may make ontogenetic development different across historical cohort and periods” (p. 587). For reasons of comprehensiveness, it should be mentioned that life span development is, according to Baltes et al., (2006), also shaped by non-normative (idiosyncratic) influences, i.e. “individual-idiosyncratic biological and environmental events” (e.g., an accident). In particular, life events that occurred to individuals prior to or during the pandemic could have – depending on personal resources and various other factors – either “inoculated” (Norris & Murrell, 1988) or steeled them, so that their well-being is quite “pandemic-resilient”, or, quite the opposite, such events might have sensitized them, so that their well-being is particularly susceptible to detrimental COVID-19 influences.

In this study, we investigate the role of the COVID-19 pandemic as such a normative history-graded influence (see also Wettstein & Wahl, 2021b) that might have caused period effects in middle-aged and older individuals’ well-being. Specifically, well-being trajectories between 2012 and 2020 will be considered as a result of normative age-graded influences, whereas specific intra-individual deviations in 2020 – the “pandemic year” - from such general trajectories will be interpreted as resulting from the pandemic as normative history-graded influence on development. Nonnormative, idiosyncratic influences are – due to their variety and their unpredictability (Baltes et al., 2006)- not easy to investigate. However, the extent of interindividual variability in “well-being reactivity” to the history-graded pandemic influence which we will also investigate is an approach to take such idiosyncratic influences – at least indirectly - into account.

Importantly, we consider well-being in this study as a multidimensional construct (Diener et al., 1999; Headey et al., 1984; Ryan & Deci, 2001); different well-being dimensions reveal differential, multidirectional change trends as people age (Diehl & Wahl, 2020; Wettstein et al., 2015). We assume that indicators of hedonic and eudaimonic well-being (Ryan & Deci, 2001) will reveal different extents of “COVID-19 reactivity” and that both pandemic-related gains and losses across different well-being domains might co-occur.

## Dimensions of Well-Being

An established distinction between two major well-being domains has been introduced by Ryan & Deci (2001). According to their theoretical framework (see also Waterman et al., 2008), the first overarching domain is labelled hedonic well-being, “which focuses on happiness and defines well-being in terms of pleasure attainment and pain avoidance” (Ryan & Deci, 2001, p. 141). The second domain, eudaimonic well-being, frequently assessed by the Scales of Psychological Well-being (Ryff & Singer, 2008; Ryff, 1989), “focuses on meaning and self-realization and defines well-being in terms of the degree to which a person is fully functioning” (Ryan & Deci, 2001, p. 141). Whereas life satisfaction as a cognitive-evaluative well-being component (Diener et al., 1999) represents an indicator of hedonic well-being, examples of eudaimonic well-being components are, according to Ryff (1989), Environmental Mastery, Personal Growth, Positive Relations With Others, and Self-Acceptance^1^.

With regard to normative age-related change in hedonic vs. eudaimonic well-being, life satisfaction as a key indicator of hedonic well-being remains remarkably stable across the adult life span, even in old and very old age, when age-related experiences of losses accumulate (Jopp et al., 2016; Wettstein et al., 2015). This phenomenon has been described as “well-being paradox” (Kunzmann et al., 2000; Schilling , 2006). Also, individuals’ hedonic well-being seems to recover from many adverse life events or health events and to return, after some time of adaptation (e.g., Schilling & Wahl 2006), to the “set point” prior to such life events (Lucas, 2007). This resilience of life satisfaction against the impact of age-related losses or of life events seems to be due to different processes. For instance, individuals adjust their standards to current conditions (“response shift”; Sprangers & Schwartz 1999). Also, according to socioemotional selectivity theory (Carstensen, 2006), individuals’ basic motivations change with advancing age and with decreasing future time perspective, shifting from goals such as autonomy or knowledge to emotionally meaningful goals such as intensifying close social relationships. As SST argues, such selectivity contributes to maintenance of high life satisfaction in late life. The theory of gerotranscendence (Tornstam, 1997) postulates that when getting older, individuals’ self-views become less narcissistic, their worldviews become less materialistic, whereas their “awareness of a cosmic dimension of reality” (p. 143) increases, which might also represent adaptive changes that contribute to maintaining high life satisfaction levels in old and very old age.

Age-related change in eudaimonic well-being is, according to Springer et al., (2011), small, accounting for only up to 4% of variation in the eudaimonic indicators (see also Clarke et al., 2000). Personal growth declines with advancing age (Clarke et al., 2000; Springer et al., 2011). Mean-level decline in environmental mastery has been reported in a longitudinal study that assessed very old individuals (Wettstein et al., 2015). Also, the cross-sectional association of environmental mastery with age is negative (Clarke et al., 2000), though not across all studies (e.g., Ryff, 1991; Ryff et al., 2004). Based on cross-sectional age group comparisons, Ryff (1989) found higher personal growth among younger and middle-aged adults than among older adults (see also Ryff 1991), whereas there were no age group differences regarding self-acceptance and positive relations with others. Still other studies found higher self-acceptance in older compared to younger age groups (e.g., Ryff 1991) as well as higher scores on positive relations with others among older individuals compared to younger adults (Ryff et al., 2004). In conclusion, empirical evidence on age patterns with regard to eudaimonic well-being is not entirely consistent. Generally, existing evidence suggests that age-related changes and age group differences across different eudaimonic well-being domains are multidirectional, revealing positive age trends for some indicators, but negative age patterns for others.

In this study, we will take the established hedonic vs. eudaimonic well-being distinction into account by including multiple well-being indicators (hedonic well-being: life satisfaction; eudaimonic well-being: a general eudaimonic indicator comprising all subscales as well as environmental mastery, personal growth, positive relations with others, self-acceptance).

## Well-Being Dimensions and Their Susceptibility to “COVID-19 Effects”

Different well-being domains differ with regard to their “reactivity” to life events (e.g., Kirsch et al., 2019) and life circumstances in general, so that they might also differ with regard to their reactivity to the pandemic.

There might thus be certain well-being indicators that are less resilient than others against the impact of the pandemic and more vulnerable to detrimental “COVID-19 effects”. For instance, the pandemic might challenge individuals’ perceived environmental mastery. From the perspective of self-determination theory (Deci & Ryan, 2000), environmental mastery can be regarded as an indicator of competence. Environmental demands have been altered due to the COVID-19 outbreak, with new demands and restrictions that have emerged (e.g. physical distancing rules; protecting oneself from infection) and the virus itself representing a highly unpredictable and only to some extent controllable environmental threat.

However, given that a crucial assumption of life-span psychology is that development in general is both multidimensional and multidirectional (Baltes et al., 2006), thus comprising gains and losses, it is possible that the COVID-19 effects on well-being are not consistently negative. Rather, the pandemic might have also promoted gains in certain well-being domains. For instance, Recchi et al., (2020) report that well-being has increased in the French population after the onset of the COVID-19 pandemic and during the lockdown compared to previous assessments, which they label “eye of the hurricane paradox”, assuming that in these serious pandemic times, many individuals “may be seeing their current condition in a more positive light than they normally would”.

Well-being increases as a consequence of the COVID-19 pandemic might have particularly occurred in domains related to the psychological need for relatedness as specified by self-determination theory (Deci & Ryan, 2000). Individuals who have been forced to reduce their social face-to-face contacts to protect themselves and others from the virus might have experienced that those remaining selective contacts became closer and more intensive during that time, resulting in a perceived gain with regard to personal relations with other people (see also Pietrzak et al., 2021). For instance, Gilligan et al. (2020) state that “the pandemic may provide opportunities for greater solidarity within families” (p. 431). The pandemic might also trigger the perception of a more limited future time perspective, which – according to socioemotional selectivity theory (Carstensen, 2006) – results in a greater motivation to intensify emotionally close relationships. Such a shift in social preferences has indeed been empirically observed as a reaction to historical events (such as the handover of Hong Kong to the People’s Republic of China; Fung et al., 1999). This process of focusing on and intensifying close relationships could also lead to more favorable perceptions of individuals with regard to their positive relations with others. Moreover, individuals might perceive that dealing with the pandemic and with the restrictions that resulted from it helped them to promote their process of personal growth (or appreciation of life; Pietrzak et al., 2021). Such positive change processes, if observable, could also be indicative of the general phenomenon of “post-traumatic growth” (Pietrzak et al., 2021).

## Empirical Evidence on the Impact of COVID-19 on Well-Being

With regard to prior evidence on COVID-19 and life satisfaction, Entringer et al., (2020) found, based on the SOEP-CoV study, that life satisfaction among German adults was on a similar level in April 2020 compared to one year ago. Similarly, Wettstein et al., (2021) found that life satisfaction slightly increased among German middle-aged and older adults between 2017 and summer 2020, and this increase corresponded to the (pre-pandemic) change in life satisfaction between 2014 and 2017. These findings suggest that life satisfaction has not been negatively affected by the onset of the pandemic. Zacher & Rudolph (2020) also found that life satisfaction did not change in a German adult sample between December 2019 and March 2020. However, according to their study, life satisfaction revealed a decline between March and May 2020. Other studies have reported lower life satisfaction in German individuals in the initial phase of the pandemic compared to pre-pandemic times such as 1–2 years prior to the pandemic onset (e.g., Bittmann 2021).

With regard to eudaimonic well-being, a study by Pellerin & Raufaste (2020) investigated change in a eudaimonic well-being indicator in France over “weeks in lockdown” in spring 2020 and observed a declining trend over the first lockdown weeks as well as a later increase in eudaimonic well-being with the approaching end of the lockdown. Based on US data, a general decline in satisfaction with social relationships between January and June 2020 has been reported (VanderWeele et al., 2021). One of the very few studies contrasting change of both hedonic vs. eudaimonic well-being assessed in parallel during the pandemic was the one by Landmann & Rohmann (2021). The authors found, based on their longitudinal assessment of a German sample between March and May 2020, that “contact restrictions impaired hedonic well-being (positive affect) but less so eudaimonic well-being (positive functioning and satisfying relationships)”.

In conclusion, empirical evidence is inconsistent with regard to how well-being and which well-being indicators changed in reaction to the pandemic outbreak. Generally, there is still a lack of studies that investigated well-being *changes* in reaction to the pandemic (see also Gaertner et al., 2021; Wettstein & Wahl, 2021a). This is particularly true with regard to studies investigating more than one well-being indicator, including eudaimonic indicators, rather than solely measures of hedonic well-being, and relying on a longitudinal study design with both pre- and peri-pandemic assessments. Specifically, following the described macro-model of developmental influences and its distinction between normative age-graded vs. normative history-graded influences on development (Baltes et al., 2006), several pre-pandemic measurement occasions are needed in order to differentiate between those two influence factors on well-being trajectories. In this study, we will therefore investigate change in multiple indicators of hedonic *and* eudaimonic well-being among middle-aged and older adults, based on three pre-pandemic measurement occasions (2012, 2015, and 2017) as well as one peri-pandemic measurement occasion that took place between June and September 2020.

## Potential Determinants of Pandemic-Related Well-Being Changes

We also assume that, as middle-aged and older adults represent a considerably heterogeneous group (Nelson & Dannefer, 1992), also with regard to well-being levels and changes (e.g., Wettstein et al., 2015), well-being trajectories related to the pandemic are not the same in size and direction for each individual (Wettstein et al., 2021). Some subgroups might thus be at a higher risk of well-being decline than others (Entringer et al., 2020; Schäfer et al., 2020; Wettstein et al., 2021). Specifically, we will control for age, gender, education, and self-rated health.

Regarding the role of age, prior research has mostly reported that older adults’ well-being seems to be less compromised or even unaltered after the onset of the pandemic (Röhr et al., 2020), whereas particularly younger or middle-aged adults report higher distress, more severe mental health problems and poorer well-being (Bäuerle et al., 2020; Benke et al., 2020; Gilan et al., 2020; Peters et al., 2020; Schlomann et al., 2021). This might be due to older adults’ higher levels of resilience, also in times of the pandemic (Gilan et al., 2020; Lind et al., 2020), and their lifetime experiences with regard to crises and how to deal with them. Also, certain consequences of the pandemic, such as remote work, short-term work and closures of schools and childcare facilities due to the pandemic had a negative impact particularly on the work and family satisfaction of working adults with young children (Möhring et al., 2020), whereas older, retired individuals were not, or at least not directly, affected by implications of the pandemic for work conditions and childcare. Pandemic-related change in eudaimonic well-being indicators such as positive relations, or relatedness satisfaction, might also vary according to individuals’ age (Schlomann et al., 2021; Schwinger et al., 2020). López et al. (2020) compared young-old and old-old individuals’ eudaimonic well-being during the lockdown in Spain and found a negative age difference in personal growth.

Another pandemic-relevant sociodemographic indicator is gender. Previous studies found lower well-being, as well as higher virus anxiety (Jungmann & Witthöft, 2020), during the pandemic among women compared to men (Benke et al., 2020; Entringer et al., 2020), particularly when middle-aged individuals are considered (Möhring et al., 2020; Wettstein et al., 2021). This gender difference among middle-aged adults might be caused to some extent by altered work conditions and challenges regarding child care because of closed schools and childcare facilities (Huebener et al., 2021), which seems to cause more child-care-related worries in women than in men (Czymara et al., 2020), as they are more often responsible for organizing childcare and home schooling than men (Ohlbrecht & Jellen, 2021).

Higher levels of education are usually associated with higher well-being scores, although this association is small (Diener et al., 1999), and higher education might also buffer the negative psychosocial consequences of the pandemic on individuals, e.g. with regard to their well-being (Benke et al., 2020; Ohlbrecht & Jellen, 2021; Traunmüller et al., 2020; Wettstein et al., 2021). For instance, individuals with higher levels of education perceive a higher subjective control over a potential infection with the Corona virus (Wettstein et al., 2020), which might contribute to less distress and fear in this group than among individuals with lower education.

Finally, a better self-rated health is a determinant of higher well-being in general (Diener et al., 1999; Kunzmann et al., 2000; Mroczek & Spiro, 2005; Smith et al., 2010). During the pandemic, the well-being of those with poorer health might be particularly compromised, as health restrictions and certain chronic diseases increase the risk of severe COVID-19 disease development when infected (Karagiannidis et al., 2020; Nachtigall et al.; Robert-Koch-Institut, 2020; Von der Lippe, 2021). Indeed, poorer self-rated health as well as more pre-existing health conditions are significant predictors of lower life satisfaction, higher anxiety, more feelings of threat, greater stress and depression, but also lower personal growth and relatedness during the Corona crisis (López et al., 2020; Petzold et al., 2020; Röhr et al., 2020; Schwinger et al., 2020; Traunmüller et al., 2020; Wettstein et al., 2020, 2021). Also, individuals whose self-rated health revealed a steeper decline between a pre-pandemic measurement occasion and March 2020, the period of the first lockdown in Germany, exhibited a steeper decrease in mental well-being during the same time interval (Peters et al., 2020).

## The Present Study

In this study, we investigate change in well-being among middle-aged and older adults across a time period of eight years, comprising both pre-pandemic measurement occasions (2012, 2015, 2017) and one peri-pandemic measurement occasion (June-September 2020). We will specify general trajectories of each well-being indicator from 2012 to 2020, assuming that such trajectories result from normative age-graded influences. In addition, as there might also be an additional impact of the pandemic as a normative history-graded influence (Baltes et al., 2006) on well-being changes, we will specify intra-individual deviations in 2020 from the general slopes which indicate the extent of pandemic-related change (for a similar analytical approach addressing the impact of COVID-19 on well-being, see Kivi et al., 2020; Wahl et al., 2022; Wettstein & Wahl, 2021b).

As pandemic-related changes in well-being can be expected to reveal a remarkable interindividual heterogeneity, we also investigate the role of age, gender, education, and self-rated health as predictors of intra-individual pandemic-related change.

## Method

### Sample

Participants were recruited in 2012 via announcements in public locations and newspaper advertisements as part of a German-American research project (Diehl et al., 2013). The target population were individuals aged 40 years and older living in private households in Germany. The resulting study sample was thus a non-probability sample. Participants were approached by researchers and students. No further inclusion or exclusion criteria were applied. Participants were offered an incentive of 10 € for their participation at each wave of the survey.

Data from four measurement occasions were used for analyses (T1 in 2012, *n* = 423; T2 in 2015, *n* = 356; T3 in 2017, *n* = 299; T4 in June-September 2020, *n* = 233). Data were collected at T1-T3 via paper-pencil questionnaires that were sent to the study participants with paid return service. At T4, data were, to a large extent (79%), collected online; 21% filled out a paper-pencil questionnaire, with the procedure being identical to the prior measurement waves. The order of questions/items in the online version and in the paperpencil version of the questionnaire was identical for the sake of comparability. The online version was first pilot-tested within the project team. It was then applied to 30 study participants in order to ensure that the online format was functional and easy to use. When comparing those who filled out a paper-pencil questionnaire at T4 with those who took part online, significant differences were found for age (*d* = 0.36), education (*d* = 0.56), general eudaimonic well-being (*d* = 0.38), environmental mastery (*d* = 0.35), and personal growth (*d* = 0.49). Individuals who took part online had on average more years of education than those who filled out the paper-pencil questionnaire, reported greater general eudaimonic well-being, greater environmental mastery and greater purpose in life. Effect sizes of these differences were, according to common classifications (Cohen, 1992), small to medium.

A sample description is provided in Table 1. Study participants were between 40 and 98 years old at baseline (*M* = 62.94 years, *SD* = 11.84 years). Individuals participated in one to four measurement occasions (*M* = 3.10, *SD* = 1.08; individuals with one study participation: *n* = 53 [12.5%]; individuals with two study participations: *n* = 68 [16.1%]; individuals with three study participations: *n* = 86 [20.2%]; individuals with four study participations: *n* = 216 [51.1%]).

**Table 1:** Sample Description (Baseline)

	2012	2015	2017	2020
	*M* ± *SD* or *n* (%)	*M* ± *SD*	*M* ± *SD*	*M* ± *SD*
Age	62.94 ± 11.84			
Female	272 (64.3%)			
Education	11.53 ± 1.96			
Self-Rated Health ^a^	2.12 ± 0.85			
Life Satisfaction	4.91 ± 1.19	5.07 ± 1.11	4.97 ± 1.10	5.07 ± 1.11
General Eudaimonic Well-Being	4.60 ± 0.51	4.56 ± 0.53	4.57 ± 0.52	4.55 ± 0.52
Environmental Mastery	4.80 ± 0.69	4.72 ± 0.78	4.72 ± 0.75	4.65 ± 0.76
Personal Growth	4.79 ± 0.77	4.67 ± 0.82	4.72 ± 0.79	4.72 ± 0.84
Positive Relations	4.33 ± 0.91	4.33 ± 0.95	4.36 ± 0.97	4.43 ± 0.96
Self-Acceptance	4.60 ± 0.93	4.65 ± 0.88	4.65 ± 0.89	4.70 ± 0.84

*Note. M* = mean; *SD* = standard deviation

^a^ Lower values indicate better self-rated health.

Among those individuals who dropped out of the study and who provided a reason for study attrition, the most frequently mentioned reasons were lack of interest and health problems. Some individuals could not be re-contacted after T1 (e.g. because of relocation or death). We found only limited evidence for selective attrition effects when comparing the 233 returners in 2020 with those 190 persons who dropped out of the study during the 8-year observational interval. All differences between these groups with regard to study variables at baseline were all of small effect size, with the exception of the age difference, which was, according to common effect size classifications (Cohen, 1992), of medium effect size (*d* = 0.59). Participants who still took part in 2020 were on average about seven years younger than participants who had dropped out of the study prior to 2020. With regard to the covariates, differences regarding education (*d* = 0.15), gender distribution (φ = 0.14) and self-rated health (*d* = 0.07) were not significant. Regarding well-being at T1, those who dropped out of the study later were not significantly different from those still participating in 2020 regarding general eudaimonic well-being (*d* = 0.03), environmental mastery (*d* = 0.03), personal growth (*d* = 0.16), and positive relations with others (*d* = 0.05), whereas their life satisfaction at baseline (*d* = 0.25) as well as their self-acceptance at baseline (*d* = 0.31) were significantly higher compared to those who still participated in the study in 2020.

#### Compliance with Ethical Standards

At every measurement occasion, individuals provided written informed consent prior to study participation. Approval for waves 3 and 4 of the study was received from the Institutional Ethics Review Board of the Faculty of Behavioral and Empirical Cultural Sciences of Heidelberg University. Waves 1–2 were approved by the Colorado State University (CSU) Institutional Review Board (IRB) protocol #10-2080H based on a formal cooperation between Heidelberg University and CSU. All individuals were informed that they could change their minds and withdraw their agreement at any time. This study was designed and organized in line with all rules and guidelines specified in the “Leitlinien zur Sicherung.

guter wissenschaftlicher Praxis”, 2019 [Good Research Practice. Guidelines for Safeguarding Good Research Practice, 2019; access via: https://wissenschaftliche-integritaet.de/en/code-of-conduct/) by the German Research Foundation.

## Measures

### Well-Being

We differentiated between hedonic and eudaimonic well-being (Ryan & Deci, 2001). Indicators representing hedonic vs. eudaimonic well-being were assessed in this study as follows.

**Hedonic well-being.** As indicator of hedonic and cognitive-evaluative well-being, life satisfaction was assessed based on the Satisfaction with Life Scale (SWLS; Diener et al., 1985). Participants answered five items (e.g., “The conditions of my life are excellent”) on a response format ranging from 1 (strongly disagree) to 7 (strongly agree). A mean score across all items was computed for each individual, with higher scores indicating greater life satisfaction. Cronbach’s α across the measurement occasions from 2012 to 2020 was 0.89, 0.91, 0.87, and 0.89. The German version of the SWLS, which we used in this study, was found to be unidimensional (see also Diener et al., 1985; Emerson et al., 2017; Pavot & Diener, 1993) and to reveal measurement invariance across seven age groups, ranging from 14 to 24 years to ≥ 75 years (Glaesmer et al., 2011). According to Wu et al. (2009), who found partial strict and partial strong longitudinal invariance of the SWLS in two samples, “the SWLS has satisfactory psychometric properties for longitudinal measurement invariance” (p. 396).

**Eudaimonic well-being**. An 18-item short-form of the Psychological Well-Being scale (Ryff & Singer, 2008; Ryff & Keyes, 1995) was used to assess eudaimonic well-being. Each subscale was assessed based on three items. According to prior findings, each of the 18 items revealed a positive and strong association with its own scale, and a 6-factor solution provides the best model fit (Clarke et al., 2000, 2001; Ryff & Keyes, 1995; Ryff & Singer, 2006). Joshanloo (2019) reported evidence in support of full metric invariance and partial scalar invariance of Psychological Well-Being (see also Joshanloo 2018), conceptualized as one factor based on the 18 items, over a period of two decades.

Following the approach of other studies (Brothers et al., 2016; Gallagher et al., 2009; Keyes et al., 2002; Springer & Hauser, 2006), and given that all six subdomains of the 18-item scale load on one higher-order factor (e.g., Linley et al., 2009), we derived an overarching indicator of general eudaimonic well-being by computing a mean score across all 18 items for each individual at each measurement occasion. This general eudaimonic well-being indicator revealed a high internal consistency across all measurement occasions (α 2012–2020:0.77, 0.78, 0.78, 0.80). Additionally, we computed mean scores for all 3-item subscales, with higher scores on each subscale indicating greater eudaimonic well-being in the respective domain. The included eudaimonic subscales (see Footnote 1) were environmental mastery (e.g., “In general, I feel I am in charge of the situation in which I live”; α 2012–2020: 0.55, 0.60, 0.61, 0.69), personal growth (e.g., “For me, life has been a continuous process of learning, changing, and growth”; α 2012–2020: 0.53, 0.54, 0.53, 0.70) positive relations with others (e.g., “People would describe me as a giving person, willing to share my time with others”; α 2012–2020: 0.52, 0.57, 0.60, 0.64), and self-acceptance (e.g., “I like most parts of my personality”; α 2012–2020: 0.78, 0.71, 0.78, 0.76). The response format for each item ranged from 1 (completely disagree) to 6 (completely agree).

**Covariates.** As covariates and predictors of pandemic-related well-being changes, we included age, gender, education, and self-rated health. Years of schooling were assessed as indicator of education. Self-rated health was measured based on a single-item (“Compared to other persons of my age, I regard my health as…”). The response format ranged from 1 (*very good*) to 6 (very poor).

### Statistical Analyses

Longitudinal multilevel regression models were computed (Hox & Kreft, 1994; Ram & Grimm, 2015) to investigate well-being trajectories between 2012 and 2020. As not all individuals took part at all four measurement occasions (mean number of observations = 3.1, *SD* = 1.1), we decided to specify linear trajectories only. Reliably estimating nonlinear (quadratic) change might require more measurement occasions per individual. Specifically, as we had a maximum three measurement occasions available (2012, 2015, 2017) to model prepandemic change, only a linear (pre-pandemic) change model could be fully specified, whereas a fully specified quadratic change model would require at least four measurement occasions (Nese, 2013).

In our models, we included a general change component between 2012 and 2020, potentially reflecting normative age-graded influences on well-being. Additionally, we specified models with such a general change plus an intra-individual deviation component from the slope in 2020 which might indicate to what extent the pandemic as a normative history-graded event altered normative age-graded trajectories of well-being. This deviation component was specified by generating a time-varying dummy variable which was set to 0 for all individuals at all measurement occasions before 2020 and which was constrained to 1 for all individuals in 2020. This approach is adopted from a study by Kivi et al. (2020), who also specified such a dummy variable in a longitudinal multilevel regression analysis to identify well-being changes that are potentially due to the pandemic outbreak (see also Wahl et al., 2022; Wettstein & Wahl, 2021b). The regression coefficient that was estimated for this dummy variable was specified as a random effect (unless this random effect had to be set to zero to ensure model convergence), as we expected considerable inter-individual differences in the extent of pandemic-related well-being changes. Models with and without the intra-individual deviation score in 2020 were compared based on their BIC scores (Kass & Raftery, 1995), on Likelihood Ratio Test (LRT) as well as based on the relative reduction in residual variance obtained by each model (R²; computed according to Xu 2003). Whenever the model including a pandemic-related change component revealed a better model fit at least according to one criterion (i.e., higher R² or lower BIC score or LRT significant and in favor of the pandemic-related change model), we interpret this as “modest” evidence in favor of this model, whereas a better model fit across two or more criteria is interpreted as “strong” evidence.

Of note, a model including a pandemic-specific deviation component might provide a better model fit (in terms of proportional reduction of residual variance, BIC comparison, and LRT) than a model that does not contain this component even if the fixed effect of this component (i.e., the mean deviation) is *not* statistically significant, although this might seem contradictory at first glance. In such a case, it might be the *random effect* of the pandemic-specific deviation (i.e., the inter-individual variation in this score), rather than the fixed effect, that contributes to a better model fit, indicating that pandemic-specific change reveals substantial interindividual variation and that omitting this interindividual variation from the model results in a poorer model fit.

Whenever the model of a well-being outcome that included the pandemic-specific deviation component resulted in a better model fit than a model without that deviation component, and whenever the random effect of the pandemic-specific deviation component could be estimated, we analyzed the role of age, gender, education, and self-rated health (at baseline) as potential predictors of intra-individual pandemic-driven change.

## Results

Findings of the multilevel longitudinal regression models with model comparisons (model without vs. with pandemic-specific deviation component) are summarized in Tables 2, 3 and 4.

**Fig. 1:**
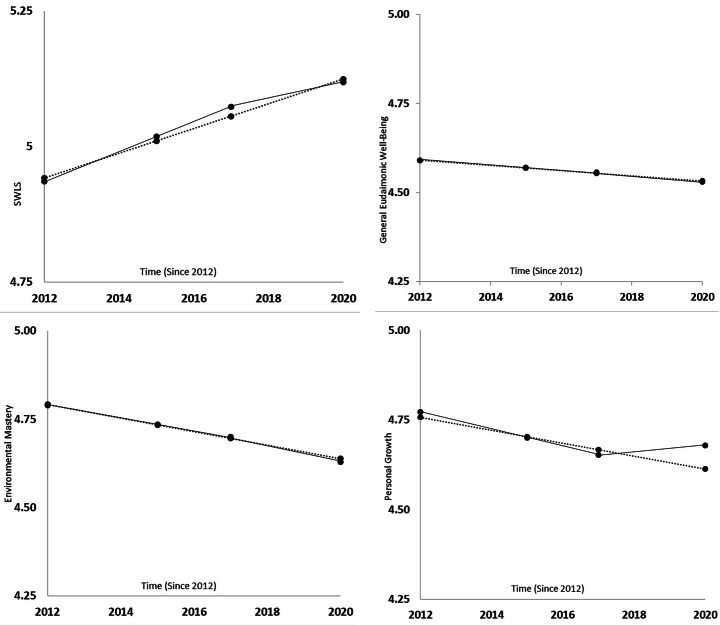
*Trajectories of Well-Being Indicators* *Note*. SWLS = Satisfaction with Life Scale. Dotted lines correspond to estimated mean-level trajectories without a pandemic-specific deviation from the general slope in 2020. Solid lines correspond to estimated mean-level trajectories including a pandemic-specific deviation component.

**Fig. 2:**
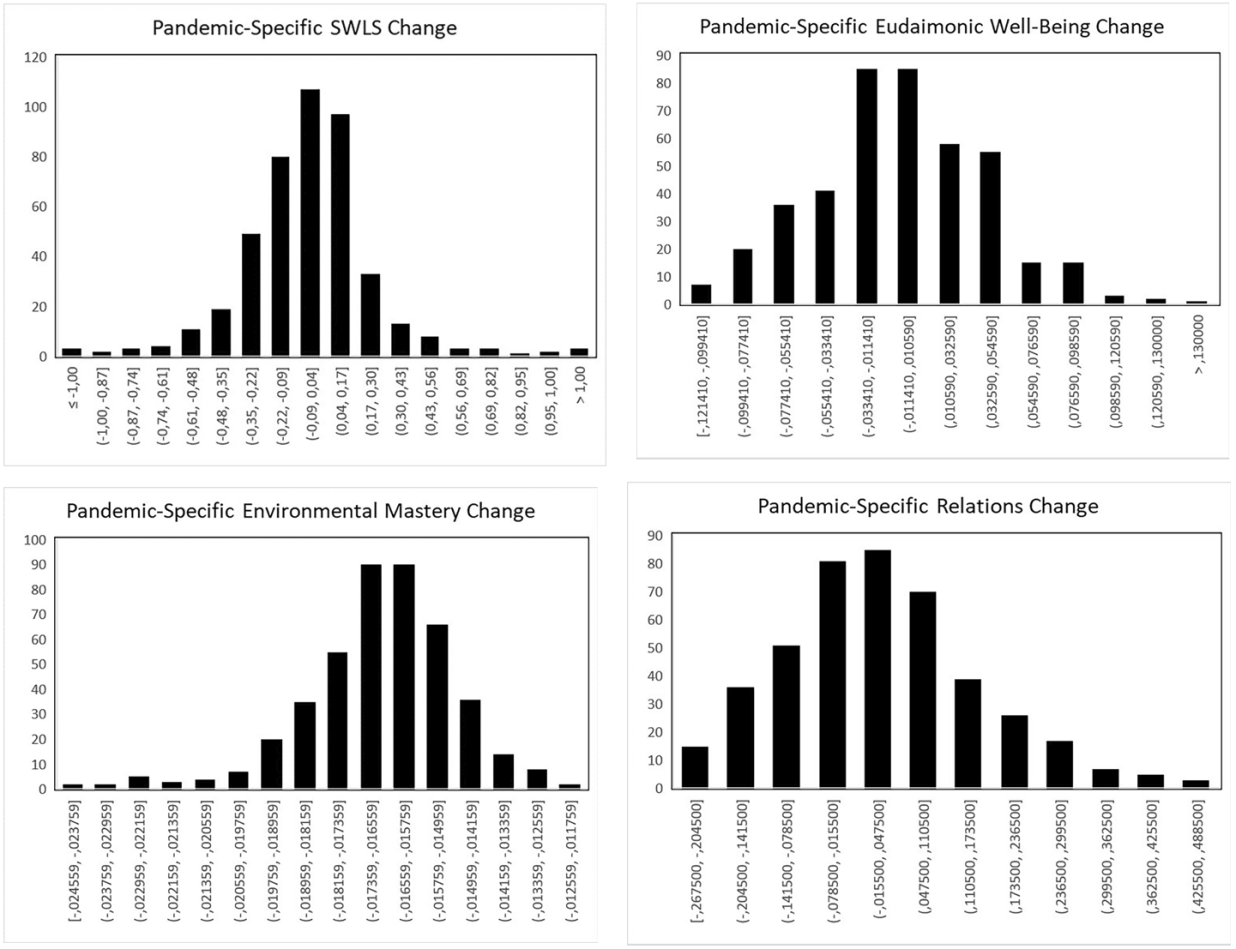
*Interindividual Differences in Pandemic-Related Deviations Across the Different Well-Being Indicators* *Note*. SWLS = Satisfaction with Life Scale.

Regarding life satisfaction (Table 2), in a model without an additional pandemic-specific deviation component, there was a small but significant mean-level increase in life satisfaction by 0.002 points per month (see Fig. 1). This estimated monthly increase in life satisfaction was the same in a model including a pandemic-specific deviation component. The additional deviation component was not significant, but the random effect of this deviation was, indicating remarkable interindividual variability in pandemic-related life satisfaction change (see Fig. 2). The BIC score was in favor of the more parsimonious model not including a pandemic-specific component, and the Likelihood Ratio Test failed to reach statistical significance (Δχ(3) = 7.1, *p* = .07), thus not favoring the pandemic-specific model. However, proportional reduction in residual variance was higher in the model including that component (*R²* = 0.23 vs. *R²* = 0.12), so that there is at least modest evidence in favor of a “COVID-19 effect” on life satisfaction trajectories.

**Table 2:** Longitudinal Multilevel Regression Models of Change in Hedonic Well-Being (Life Satisfaction)

Model Estimates	Life satisfaction linear change	Life satisfaction linear change plus pandemic-related change
Fixed Regression Coefficients:		
Intercept [*SE*]	4.942*** [0.055]	4.936*** [0.057]
Linear slope 2012–2020 [*SE*]	0.002** [0.001]	0.002*[0.001]
Pandemic-specific deviation 2020 [*SE*]		-0.038 [0.078]
Random Variances:		
Variance Intercept [*SE*]	1.043*** [0.090]	1.110*** [0.097]
Variance Linear Slope [*SE*]	0.000** [0.000]	0.000** [0.000]
Variance Pandemic-Specific deviation [*SE*]		0.431** [0.181]
Residual Variance [*SE*]	0.344*** [0.020]	0.300*** [0.023]
BIC	3,325.5	3,342.5
*R*²	0.12	0.23

*Note*. Time unit is months (since 2012). *R*² was computed according to Xu (2003). Covariances between random effects are not reported in the table.

* *p* < .05; ** *p* < .01; *** *p* < .001

**Table 3:** Longitudinal Multilevel Regression Models of Change in General Eudaimonic Well-Being

Model Estimates	Eudaimonic well-being linear change	Eudaimonic well-being linear change plus pandemic-related change
Fixed Regression Coefficients:		
Intercept [*SE*]	4.591*** [0.024]	4.590*** [0.025]
Linear slope 2012–2020 [*SE*]	-0.001* [0.000]	-0.001 [0.000]
Pandemic-specific deviation 2020 [*SE*]		-0.005 [0.033]
Random Variances:		
Variance Intercept [*SE*]	0.196*** [0.018]	0.195*** [0018]
Variance Linear Slope [*SE*]	0.000** [0.000]	0.000* [0.000]
Variance Pandemic-Specific deviation [*SE*]		0.008 [0.038]
Residual Variance [*SE*]	0.073*** [0.004]	0.072*** [0.006]
BIC	1,316.9	1,338.6
*R*²	0.11	0.13

*Note*. Time unit is months (since 2012). *R*² was computed according to Xu (2003). Covariances between random effects are not reported in the table.

* *p* < .05; ** *p* < .01; *** *p* < .001

**Table 4:** Longitudinal Multilevel Regression Models of Change in Domains of Eudaimonic Well-Being (Environmental Mastery, Personal Growth, Positive Relations with Others, Self-Acceptance)

Model Estimates	Environmental mastery linear change	Environmental mastery linear change plus pandemic-related change	Personal growth linear change	Personal growth linear change plus pandemic-related change	Positive relations linear change	Positive relations linear change plus pandemic-related change	Self-acceptance linear change	Self-Acceptance linear change plus pandemic-related change
Fixed Regression Coefficients:								
Intercept [*SE*]	4.792*** [0.033]	4.789*** [0.033]	4.757*** [0.036]	4.772*** [0.037]	4.310*** [0.044]	4.313*** [0.043]	4.608*** [0.043]	4.603*** [0.044]
Linear slope 2012–2020 [*SE*]	-0.002*** [0.000]	-0.001* [0.001]	-0.001** [0.000]	-0.002** [0.001]	0.001* [0.001]	0.001 [0.001]	0.001** [0.000]	0.002* [0.001]
Pandemic-specific slope 2017–2020 [*SE*]		-0.017 [0.057]		0.099 [0.063]		0.018 [0.060]		-0.035 [0.061]
Random Variances:								
Variance Intercept [*SE*]	0.293*** [0.033]	0.282*** [0.036]	0.355*** [0.039]	0.357*** [0.039]	0.634*** [0.056]	0.614*** [0.058]	0.603*** [0.055]	0.603*** [0.055]
Variance Linear Slope [*SE*]	0.000 [0.000]	0.000 [0.000]	0.000* [0.000]	0.000* [0.000]	0.000*** [0.000]	0.000* [0.000]	0.000 [0.000]	0.000* [0.000]
Variance Pandemic-Specific Slope [*SE*]		0.033 [0.112]				0.041 [0.222]		
Residual Variance [*SE*]	0.217*** [0.013]	0.213*** [0.017]	0.259*** [0.015]	0.258*** [0.015]	0.228*** [0.013]	0.222*** [0.018]	0.243*** [0.014]	0.242*** [0.014]
BIC	2,458.5	2,480.0	2,714.9	2,718.4	2,832.8	2,850.6	2,768.8	2,774.5
*R*²	0.04	0.06	0.08	0.08	0.14	0.17	0.07	0.07

*Note*. Time unit is months (since 2012). *R*² was computed according to Xu (2003). Covariances between random effects are not reported in the table

* *p* < .05; ** *p* < .01; *** *p* < .001

With respect to the general eudaimonic well-being indicator (see Table 3), there was a significant mean-level decline in a model without a pandemic-specific deviation component. This decline was no longer significant when adding the deviation component, which was not significant. The BIC score was in favor of the more parsimonious model without a pandemic-related deviation component, and the Likelihood Ratio Test indicated no significant model difference (Δχ(3) = 2.4, *p* = .49). However, the proportional reduction in residual variance was again – though only slightly - higher in the pandemic-related change model (R² = 0.13 vs. R² = 0.11), which can be seen as modest evidence in favor of this model. Yet both models provided very similar trajectory estimates (see Fig. 1).

The eudaimonic indicator *environmental mastery* revealed, in both models, a significant mean-level decline (see Table 4). In the model including a pandemic-specific deviation, this deviation component was not significant. The BIC comparison was in favor of the more parsimonious model, and the Likelihood Ratio Test indicated no difference between models (Δχ(3) = 2.7, *p* = .44), whereas (slightly) more variance in the outcome could be accounted for when the pandemic-specific component was included (*R²* = 0.06 vs. *R²* = 0.04). There is thus modest evidence for a small pandemic effect on environmental mastery trajectories. However, as illustrated in Fig. 1, estimated mean-level trajectories did again not substantially differ in both models.

The eudaimonic indicator *personal growth* revealed a significant mean-level decline in both models. In the model including the pandemic-specific deviation in 2020, this deviation component was not statistically significant. As the model fit in terms of the BIC score was better for the model including no pandemic-specific effect and *R²* scores were identical for both models (*R²*= 0.08)^2^, there seems to be no “COVID-19 effect” on the eudaimonic indicator personal growth.

There was a significant mean-level increase in *positive relations with others*, when no pandemic-specific component was specified. This increase was no longer significant when the pandemic-related deviation was included. The deviation component itself was not significant. *R²* was higher in the model including the pandemic-component (*R²*= 0.17 vs. *R²* = 0.14), whereas the BIC score was better in the other model, and the Likelihood Ratio Test failed to reach statistical significance (Δχ(3) = 6.4, *p* = .09). In conclusion, model comparisons indicate at least modest evidence in favor of the pandemic-related model.

Finally, *self-acceptance* significantly increased over time in both models. In the model including a pandemic-related deviation component, this component was negative, but not significant. Both models did not differ in proportions of variance accounted for (*R²* = 0.07) ^2^, but the BIC model fit was better for the more parsimonious model, so that we rejected the assumption of a COVID-19 effect on self-acceptance.

### Inter-Individual Variability in Pandemic-Related Well-Being Trajectories

The random effect of the pandemic-specific deviation component, indicating the extent of interindividual differences in pandemic-related change, reached statistical significance for life satisfaction only. However, statistical tests were based on Wald tests; according to Hox (2013) “Testing variances using the standard error is generally not a very accurate approach, because variances do not have a normal distribution”. Even if not significant, the random effects could thus indicate substantial interindividual differences. We indeed found such interindividual differences in pandemic-specific deviations also for the other well-being indicators (with the exception of personal growth and self-acceptance for which the respective random components could not be estimated, see Footnote 2). With regard to life satisfaction, for more than 55% of the sample the pandemic-specific deviation was negative (see Fig. 2) and thus indicated a shift toward lower life satisfaction; however, there was also a substantial subgroup of individuals whose life satisfaction (slightly) increased. With regard to general eudaimonic well-being, a pandemic-related shifted toward lower scores was estimated for about 56% of the sample, whereas this shift was positive for the remaining 44%. For environmental mastery, the estimated pandemic-specific deviation score was negative for all individuals, indicating a shift toward lower environmental mastery scores. However, the size of the shift ranged between – 0.025 and − 0.017, which corresponds to a small effect and to limited interindividual heterogeneity with regard to this effect. Regarding positive relations with others, the pattern was more heterogeneous. Specifically, although the fixed component corresponding to the mean pandemic-specific deviation score was positive (but statistically not significant), the individual deviation score was a negative estimate for more than 47% of the sample. It thus seems that approximately half of the sample experience more positive relations with others at the time of the pandemic, whereas the other half perceives less positive relations.

### Determinants of Pandemic-Related Well-Being Trajectories

Regarding predictors of the pandemic-specific deviations across the different well-being domains, the only significant predictor of that deviation in life satisfaction was self-rated health (β = -0.23, *p* < .05). Each point higher on self-rated health, indicating poorer self-rated health, was associated with a by 0.23 points more negative pandemic-specific deviation in life satisfaction. Pandemic-specific change toward lower life satisfaction was thus particularly pronounced among those who reported poorer health at baseline in 2012.

For general eudaimoinc well-being, environmental mastery and positive relations with others, none of the predictors of the pandemic-specific deviation component reached statistical significance. Although an older age tended to be related with a pandemic-related shift toward lower environmental mastery, this association failed to reach statistical significance (β = -0.07, *p* = .06). Similarly, more years of education were associated with a pandemic-related shift toward more positive relations, but this association did also not reach statistical significance (β = 0.04, *p* = .09). For personal growth and self-acceptance, the random effect of the pandemic-specific deviation could not be estimated and had to be restricted to zero, so that predictors of inter-individual differences in that deviation could not be investigated.

## Discussion

In this study, we investigated change in hedonic vs. eudaimonic well-being between 2012 and 2020 in middle-aged and older adults. By specifying a COVID-19-related deviation score for the year 2020, we differentiated, following the central assumptions of the macro-model of developmental influences (Baltes et al., 2006), between normative-age graded change from potentially normative history-graded change as elicited by the pandemic outbreak in 2020.

### Normative Age-Related Change in Hedonic and Eudaimonic Well-Being

Some normative age-related changes in hedonic as well as eudaimonic well-being were observed. Notably, most of these changes revealed a remarkable interindividual variability as indicated by significant random effects of the slope components. This finding underlines the “aged heterogeneity” (Nelson & Dannefer, 1992) of middle-aged and older adults with regard to developmental domains such as well-being and its change with advancing age.

Life satisfaction revealed a small but significant overall mean-level increase between 2012 and 2020. This trend might reflect the complex, nonlinear age pattern of life satisfaction – or of well-being in general (Blanchflower & Oswald, 2004), with life satisfaction levels increasing between midlife and early-old age, but decreasing thereafter (Mroczek & Spiro, 2005; Wettstein & Spuling, 2019). General eudaimonic well-being decreased over time, although this decline was no longer significant when taking an additional COVID-19 related change component into account. Declining trends were also found for the domain-specific eudaimonic indicators environmental mastery, and personal growth, which is in line with other study findings (e.g., Clarke et al., 2000; Springer et al., 2011), whereas self-acceptance increased (Ryff, 1991). Only positive relations with others revealed a favorable change trend over time, i.e., a mean-level increase (which was, however, no longer significant when a COVID-19 related change component was additionally specified and included in the model). Higher scores on positive relations with others among older compared to middle-aged adults have also been reported by Ryff et al., (2004). Generally, and in line with prior empirical evidence (Springer et al., 2011), age-related changes in eudaimonic well-being were small.

### COVID-19-Related Change in Hedonic and Eudaimonic Well-Being

Although the mean-level effects of the pandemic-specific deviation component were nonsignificant across all well-being outcomes, there was substantial interindividual variability in this component for most well-being components, and model fit (in terms of proportional reduction in residual variance) was better when including the pandemic-specific component for all well-being indicators except personal growth and self-acceptance. There is thus some evidence in favor of pandemic-related well-being change, although this change is, across all well-being components, small when considered as a mean-level effect and should better be described in terms of inter-individual differences. Specifically, for life satisfaction and general eudaimonic well-being, a bit more than 50% revealed a negative pandemic-specific deviation component, whereas the change estimate was positive (or about 0) for all others. Similarly, for 47% of the sample, the estimated pandemic-specific deviation in positive relations was negative, whereas it was positive (or close to 0) for the majority. Only for environmental mastery, there was a more uniform picture, with all individuals revealing a negative pandemic-related deviation, though this deviation was small in size for most individuals.

Interestingly, there were certain well-being indicators – namely personal growth and self-acceptance – that remained apparently unaffected by the pandemic, hence showing no improvement in model fit when including a pandemic-specific change component. Generally, this finding in line with our assumption that the susceptibility of well-being to “COVID-19 effects” might be domain-specific. Decline in life satisfaction due to the pandemic, which we found for some, though not all individuals, has also been reported by other studies (Bittmann, 2021; Schlomann et al., under review; Schwinger et al., 2020; Zacher & Rudolph, 2020), although there are also studies reporting stability, or even slight increase, in life satisfaction despite COVID-19 (e.g., Entringer et al., 2020; Wettstein et al., 2021).

We assumed that environmental mastery, which represents, from the perspective of self-determination theory (Deci & Ryan, 2000), a component of competence, might be particularly challenged during COVID-19. Specifically, the dynamic situation of changing rules of restrictions (e.g., with regard to face-to-face contacts) and the unpredictable character of COVID-19 infection rates and their development might elicit feelings of not having sufficient control over environmental demands and threats. However, the mean pandemic-specific deviation in environmental mastery was – just like for all other well-being indicators – not significant and of small effect size, so that the pandemic does not seem to have caused dramatic decreases in environmental mastery, or at least not at the study’s time of assessment in summer/autumn 2020.

For many of the well-being domains investigated, we could identify substantial subgroups whose well-being even improved between 2017 and 2020. That is, the “eye of the hurricane paradox” postulated by Recchi et al., (2020) who observed a well-being increase in France after the pandemic onset, and the phenomenon of COVID-19-associated posttraumatic growth (Pietrzak et al., 2021) were, in our study, to some extent confirmed. For instance, the pandemic might have triggered – among some individuals - more favorable perceptions with regard to positive relations with others, potentially because the lockdown and physical distancing rules have forced individuals to some extent to be more selective with regard to their relationships and to focus on contacts with their most meaningful social partners. These contacts and relationships might in turn have improved, and with contacts being restricted due to COVID-19, individuals might appreciate their remaining contacts even more. Also from the perspective of socio-emotional selectivity theory (Carstensen, 2006), historical events such as the pandemic might affect individuals’ perceptions of their time perspective and result in a higher motivation to intensify emotionally meaningful social relationships (e.g., Fung et al., 1999). Generally, the pandemic might thus not have solely negative social consequences.

Pellerin & Raufaste (2020) observed an increase in eudaimonic well-being with the approaching end of the lockdown. Our peri-pandemic measurement occasion took place after the first “hard lockdown” in Germany, between June and September 2020, when infection rates were rather low and when many governmental restrictions were already released. Many individuals might have been grateful at that point in time for the opportunity to meet meaningful others again without major restrictions, and in consequence they might have rated their relations with others, but also other well-being domains, more positively.

Generally, having no assessment available at the time of the “hard” lockdown, we cannot further investigate changes in well-being that occurred during the pandemic. Further research and “well-being monitoring” is needed, as the pandemic is still ongoing and its effects on well-being, but also processes of adaptation and well-being recovery, might come with some delay.

Finally, personal growth and self-acceptance were the only well-being indicators which did not reveal any pandemic-related shift, neither toward the positive nor toward the negative. Change in these indicators over eight years was thus apparently solely driven by maturational, age-graded influences (Clarke et al., 2000; Ryff, 1989, 1991; Springer & Hauser, 2006), whereas the pandemic might not have affected how individuals perceive their personal growth or to what extent they accept themselves. Our finding of no “COVID-19 effects” on these two eudaimonic indicators can also be seen in line with Landmann and Rohmann (2021) who report that the pandemic and COVID-19-related contact restrictions affected hedonic well-being to a larger extent than eudaimonic well-being. However, our study findings support this conclusion only for two eudaimonic indicators and also in comparison to only one available hedonic indicator, namely life satisfaction. Also, there might be methodological causes for the non-improvement in model fit after specifying a pandemic-related change component for personal growth and self-acceptance, as interindividual variation in intra-individual pandemic-related change had to be restricted to 0 for these two indicators, which means that the strict constraint was set that the extent of pandemic-related change in these indicators does not vary between individuals.

In conclusion, overall mean-level effects of pandemic-related changes in hedonic and eudaimonic well-being are small and, for most indicators, remarkably heterogeneous. It seems that the pandemic has – at least in summer and autumn 2020 – not elicited a general, dedifferentiated decrease across all well-being indicators considered. Rather, the multidimensionality and multidirectionality of well-being persists during the pandemic.

### Determinants of Pandemic-Related Changes

As already pointed out, pandemic-related changes in well-being revealed some interindividual heterogeneity. To identify individuals who are at highest risk for well-being declines after the onset of the pandemic, we included age, gender, education, and self-rated health as predictors of pandemic-related changes.

Only two predictor effects reached statistical significance. Pandemic-related decline in life satisfaction was steeper among those with poorer self-rated health. This is in line with other studies reporting that restricted health is risk factor for lower life satisfaction and compromised well-being during the pandemic (López et al., 2020; Peters et al., 2020; Petzold et al., 2020; Röhr et al., 2020; Schwinger et al., 2020; Traunmüller et al., 2020; Wettstein et al., 2021). Individuals with poorer health and pre-existing health conditions have a higher risk of severe COVID-19 progression when infected with the virus (Karagiannidis et al., 2020; Nachtigall et al.; Robert-Koch-Institut, 2020; Von der Lippe, 2021), which might explain why their well-being is particularly compromised during the pandemic. Indeed, persons with poorer health or who worry more about their health also tend to feel more threatened by the pandemic (Jungmann & Witthöft, 2020; Wettstein et al., 2020), which might reflect their objectively greater COVID-19 health risks, but also in consequence compromise their well-being more compared to individuals with better health and fewer health-related worries.

Age did not significantly predict pandemic-related change in any well-being domain. Obviously, an older age comes with heightened COVID-19 health risks, but not necessarily with more negative psychosocial consequences, e.g., in terms of well-being, which underlines the resilience of older adults, also and particularly in times of the pandemic (Lind et al., 2020) and which is in line with other evidence regarding the impact of the pandemic on older individuals’ psychosocial functioning (Röhr et al., 2020; Wettstein & Wahl, 2021a).

According to our findings, socio-demographic indicators such as age, but also education or gender, thus do not seem to determine to what extent individuals experience pandemic-related well-being changes. Rather, psychological resources, such as sense of coherence (Schäfer et al., 2020) or resilience (Röhr et al., 2020), but also material resources (subjective standard of living; Wettstein et al., 2021), as well as individual appraisals, such as the extent to which individuals feel threatened by the pandemic and to which they perceive control over a potential infection (Brose et al., 2020; Wettstein et al., 2020, 2021; Whitehead, 2021; Zacher & Rudolph, 2020) might be important antecedents of pandemic-related well-being dynamics which requires further investigation by future studies. This is also true with regard to the nonnormative (idiosyncratic) developmental influences as specified in the macro-model of developmental influences (Baltes et al., 2006): Individual life events prior to the pandemic might have steeled or inoculated some individuals for the time of the pandemic, but sensitized others, which needs to be investigated by future research.

## Limitations

Among the strengths of this study are the availability of a longitudinal and multi-dimensional well-being assessment comprising both hedonic and eudaimonic aspects. Also, given that pre-pandemic measurement occasions were available in addition to one peri-pandemic occasion, we did not have to rely, unlike other studies, on retrospective reports which are potentially biased (Hipp et al., 2020), and we were able to differentiate between maturational-age-graded changes in well-being and history-graded change as potentially caused by the pandemic.

However, one of the study’s limitations is that only one hedonic well-being indicator was assessed, namely life satisfaction, so that we could not track pandemic-related changes in other hedonic domains such as affective well-being. Also, we had to exclude two eudaimonic indicators (autonomy, purpose in life) from the analyses due to their insufficient psychometric properties. Potential peri-pandemic well-being changes in reaction to the dynamics of the pandemic, including temporary rises and decreases in infection rates or increasing vaccination rates, could not be addressed in this study as only one peri-pandemic measurement occasion was available.

Some of the eudaimonic indicators, each of them assessed based on only three items, revealed low internal consistency (Cronbach’s α) at some measurement occasions. Moreover, although there is evidence in support of a 6-factor solution of Psychological Well-Being, also based on the 18-item short-form which was used in this study (Clarke et al., 2000, 2001; Ryff & Keyes, 1995; Ryff & Singer, 2006), the scales of Psychological Well-Being have also been criticized because of very strong overlap among the dimensions (e.g., Springer & Hauser, 2006). Also, there is a lack of studies addressing longitudinal invariance of the domains of Psychological Well-Being, so that we cannot entirely rule out that changes we observed might to some extent be due to a lack of invariance over time.

All our model comparisons which resulted in a better fit for those models containing a pandemic-specific change component only in terms of proportional reduction of residual variance (R²; Xu 2003), but not in terms of other criteria (BIC, LRT). Thus, for all model comparisons resulting in a decision in favor of the pandemic-related models, their superiority in terms of model fit was modest. Moreover, it is controversial how to compute and interpret R² in longitudinal multilevel regression models, and available R² measures can lead to different estimates and have several shortcomings (Rights & Sterba, 2019), therefore our findings need to be interpreted cautiously.

Changes in well-being between 2017 and 2020 might to some extent be biased by the change of assessment mode from paper-pencil questionnaire in 2017 to an online questionnaire for about 80% of the study participants in 2020. However, to avoid selection bias, participants who did not want to take part in the online survey in 2020 were not excluded from participation, but received a paper-pencil questionnaire. When comparing the well-being scores in 2020 of those who participated online with those who filled out a paper-pencil questionnaire, no significant differences were found for life satisfaction, positive relations, and self-acceptance. Significant differences were found for general eudaimonic well-being, environmental mastery, and personal growth, but these differences were in a small to medium effect size range. It is thus unlikely that the change in assessment mode in 2020 has severely affected estimates of well-being changes. Moreover, according to Weigold et al., (2013), methods of paper-and-pencil vs. Internet data collection are generally equivalent.

All well-being outcomes, as well as the predictors, were based on self-reports. While self-reports are the common assessment format for subjective well-being, it would have been desirable to have additional objective predictors available – e.g. objective health data in addition to subjective health (although the predictive validity of subjective health, e.g. in terms of mortality risk is well established; e.g. DeSalvo et al., 2006; Graf & Hicks Patrick, 2016; Idler & Benyamini, 1997).

Of the originally 423 study participants, only 233 still took part in the fourth measurement occasion in 2020. Selective dropout might thus have biased our results and change estimates to some extent. However, comparing the dropout sample and the sample of non-dropouts, only their difference in baseline age was of medium effect size, whereas all other group differences were of small effect size, and only two differences in well-being outcomes (life satisfaction and self-acceptance) were statistically significant. Severely biased estimates due to selective study attrition in our study are thus unlikely. Also, one major advantage of longitudinal multilevel models is that estimations are based on all available information – including observations from those who dropped out already after the first measurement occasion.

Intra-individual deviations in 2020 from general 8-year normative well-being changes might not necessarily be due to the pandemic. However, we had no “pandemic-free” control group available – which is due to the nature of the pandemic as a universal and *normative* history-graded event. Moreover, from our point of view, there are no other convincing explanations, such as alternative period effects in that time period, for the deviations observed. Therefore, we consider the assumption of a “COVID-19 effect”, at least on some of the well-being indicators investigated, as the most plausible explanation.

## Conclusion

Based on 8-year longitudinal data of a sample comprising middle-aged and older adults, we observed pandemic-related change patterns in hedonic and eudaimonic well-being that reflected the general multidimensional and multidirectional character of well-being. Although mean-level pandemic-related changes were consistently nonsignificant throughout all well-being indicators considered, these changes revealed, for most indicators, remarkable amounts of inter-individual heterogeneity. Also, the inclusion of this change component revealed, again for most well-being indicators, a better model fit than when no “COVID-19 effect” was specified.

According to our findings, COVID-19 might have the potential to harm as well as to promote individuals’ well-being. To prevent detrimental effects of COVID-19 on well-being and to optimally exploit its potential for promoting certain well-being aspects, more research is needed that examines the *mechanisms* via which the pandemic affects well-being and that identifies the subgroups that are most vulnerable for pandemic-related well-being declines.

## Footnotes

1 The two remaining scales of the Psychological Well-Being construct by Ryff (1989), autonomy and purpose in life, were not included in this study as they revealed poor psychometric properties (Cronbach’s α < 0.50 at two or more measurement occasions). These two scales are therefore not further discussed here. The items of these two excluded scales were, however, included when a composite score of general eudaimonic well-being was computed by calculating a mean score across all items.

2 To achieve model convergence, the random component of the pandemic-related deviation score had to be omitted. Therefore, the model without the pandemic-related component and the model with this component had the same number of random effects to be estimated so that we could not compute the Likelihood Ratio Test.
